# Supplementary material for: How much does it cost to implement the Baby-Friendly Hospital Initiative training step in the United States and Mexico?
Source: PLoS One. 2022 Sep 28;17(9):e0273179. doi: 10.1371/journal.pone.0273179 (PMC9518892; doi:10.1371/journal.pone.0273179)
Supplement: S2 Fig — (DOCX) [file pone.0273179.s002.docx]

**S2 Figure. Births per day in 2018 and 2019 in Mexico from birth certificates issued from the Mexican Civil Registry Offices** [44]**.**

^a^n=2,162,432 (n=125 were removed due to missing values or data entry errors). ^b^n=2,092,142 (n=96 were removed due to missing values or data entry errors).
