# Supplementary material for: How much does it cost to implement the Baby-Friendly Hospital Initiative training step in the United States and Mexico?
Source: PLoS One. 2022 Sep 28;17(9):e0273179. doi: 10.1371/journal.pone.0273179 (PMC9518892; doi:10.1371/journal.pone.0273179)
Supplement: S1 Table — (DOCX) [file pone.0273179.s003.docx]

**S1 Table. Sensitivity analyses assuming uniform weekly births and double the staff for total hospital births and cost per birth (in USD) for BFHI training in the United States and Mexico.**

| **United States** | **Hospital level 1^a^** | | **Hospital level 2^a^** | | **Hospital level 3^a^** | |
| --- | --- | --- | --- | --- | --- | --- |
|  | <800 annual births  (n=144) | ≥800 annual births  (n=168) | <1300 annual births  (n=297) | ≥1300 annual births  (n=340) | <2800 annual births  (n=221) | ≥2800 annual births  (n=231) |
| Total annual births | 91,081 | 255,582 | 265,277 | 826,332 | 392,290 | 1,049,076 |
| **Uniform Weekly Births^b^** | | | | | | |
| Mean births per day (SD) | 1.73 (0.24) | 4.17 (2.76) | 2.45 (0.63) | 6.66 (3.39) | 4.86 (1.7) | 12.44 (4.9) |
| Mean cost per birth with minimal training (SD)^c^ | 75.21 (5.74) | 8.67 (2.82) | 11.93 (4.32) | 5.60 (1.65) | 22.08 (2.23) | 9.33 (0.79) |
| Mean cost per birth with comprehensive training (SD)^c^ | 91.67 (5.93) | 13.02 (3.03) | 17.46 (4.52) | 8.28 (1.74) | 26.09 (2.41) | 11.54 (0.83) |
| **Double Staff^b^** | | | | | | |
| Mean births per day (SD) | 1.73 (0.24) | 4.17 (2.76) | 2.45 (0.63) | 6.66 (3.39) | 4.86 (1.7) | 12.44 (4.9) |
| Mean cost per birth with minimal training (SD)^c^ | 144.81 (5.75) | 15.03 (2.84) | 20.17 (4.29) | 9.83 (1.65) | 42.53 (2.24) | 18.00 (0.8) |
| Mean cost per birth with comprehensive training (SD)^c^ | 176.34 (5.92) | 22.92 (2.99) | 30.36 (4.45) | 14.89 (1.77) | 50.04 (2.43) | 22.18 (0.83) |
| **Mexico** | **Hospitals ≤2000 annual births**  (n=73) | **Hospitals >2000 annual births**  (n=81) |  |  |  |  |
| Total annual births | 76,217 | 307,969 |  |  |  |  |
| **Uniform Weekly Births^b^** | | | | | | |
| Mean births per day (SD) | 2.86 (1.22) | 10.42 (6.17) |  |  |  |  |
| Mean cost per birth (SD) | 2.59 (3.61) | 1.10 (1.16) |  |  |  |  |
| **Double Staff^b^** | | | | | | |
| Mean births per day (SD) | 2.86 (1.22) | 10.42 (6.17) |  |  |  |  |
| Mean cost per birth (SD) | 2.72 (3.60) | 1.23 (1.16) |  |  |  |  |

^a^Level 1 of obstetric care provided services for uncomplicated maternity and newborn cases; level 2 provided service for all uncomplicated and most complicated cases; and level 3 provided services for all serious illnesses and abnormalities. ^b^Uniform distribution of weekly births assumed (births occurred in equal amounts on all 365 days of the year, not just weekends); Double Staff assumed births were concentrated on the weekdays (255 days of the year) and, as a high end estimate, staff were multiplied by two. ^c^Wage replacement and direct training costs were calculated using the minimal and comprehensive number of training hours, respectively, from Table 5.
